# The bZIP Transcription Factor Family in Adzuki Bean (*Vigna Angularis*): Genome-Wide Identification, Evolution, and Expression Under Abiotic Stress During the Bud Stage

**DOI:** 10.3389/fgene.2022.847612

**Published:** 2022-04-25

**Authors:** Zhengong Yin, Xianxin Meng, Yifan Guo, Shuhong Wei, Yongcai Lai, Qiang Wang

**Affiliations:** Crop Resources Institute of Heilongjiang Academy of Agricultural Sciences Harbin, Heilongjiang, China

**Keywords:** adzuki bean, bZIP members, analysis, relative expression, bud stage, abiotic stress

## Abstract

Adzuki bean (*Vigna angularis*) is an important dietary legume crop that was first cultivated and domesticated in Asia. Currently, little is known concerning the evolution and expression patterns of the basic leucine zipper (bZIP) family transcription factors in the adzuki bean. Through the PFAM search, 72 bZIP members of adzuki bean (VabZIP) were identified from the reference genome. Most of them were located on 11 chromosomes and seven on an unknown chromosome. A comprehensive analysis, including evolutionary, motifs, gene structure, *cis*-elements, and collinearity was performed to identify VabZIP members. The subcellular localization results showed VabZIPs might locate on the nuclear. Quantitative real-time PCR (qRT-PCR) analysis of the relative expression of VabZIPs in different tissues at the bud stage revealed that VabZIPs had a tissue-specific expression pattern, and its expression was influenced by abiotic stress. These characteristics of *VabZIPs* provide insights for future research aimed at developing interventions to improve abiotic stress resistance*.*

## Introduction

Transcription factors (TFs), which constitute approximately 8% of the protein-encoding regulators in eukaryotic genomes, are critical transcriptional regulatory factors (Pruneda-Paz et al., 2014). Therefore, functional characterization of transcription factors (TFs) is critical for understanding transcriptional regulatory networks and biological processes (Liu et al., 2014). The basic leucine zipper (bZIP) family is one of the largest and most diverse TF families (Pérez-Rodríguez et al., 2010). The bZIP domain is highly conserved and contains two structural features located on a contiguous α-helix i.e., the leucine zipper composed of several heptad repeats of Leu or other bulky hydrophobic amino acids for dimerization specificity, and the N-x7-R/K-x9 domain for specific binding (Jakoby et al., 2002; Lee et al., 2006; Nijhawan et al., 2008). Apart from the bZIP domain, several domains of the bZIP family have been found to function as transcriptional activators (Liao et al., 2008). To bind DNA, half of the basic region in the N-terminal binds double-stranded DNA, and half of the Leu zipper in the C-terminal undergoes dimerization, leading to the formation of a superimposed coiled structure (Ellenberger et al., 1992).

Members of the bZIP transcription factor family are involved in the regulation of growth and developmental processes such as seed germination, embryogenesis, flower and vascular development, hormonal control, and senescence (Jakoby et al., 2002; Schütze et al., 2008; Toh et al., 2012; Sornaraj et al., 2016). Overexpression of *OsbZIP23*, a member of bZIP in rice (*Oryza sativa*), rescued the pre-harvest budding phenotype and the decrease in expression of genes associated with ABA signaling in transgenic plants (Song et al., 2020). *CAREB1*, an important trans-acting factor of bZIP members, was found to regulate somatic embryogenesis in carrot (*Daucus carota*) (Guan et al., 2009). Eleven *TabZIP* genes in wheat (*Triticum aestivum*) were highly expressed in anthers, suggesting that they were involved in flower development (Li D. et al., 2015). In *Arabidopsis*, a bZIP transcription factor that control monopteros (MP) output and modulate vascular gene expression (Smit et al., 2020). The bZIP Transcription factor PERIANTHIA interacts with a variety of developmental pathways, including light and plant hormones, both of which participate in meristem formation (Maier et al., 2011). Furthermore, the bZIP members regulate response to abiotic/biotic stresses such as drought, salt, hypoxia, cold, pests, and diseases (Uno et al., 2000; Shimizu et al., 2005; Zander et al., 2012; Alves et al., 2013; E et al., 2014; Amorim et al., 2017). *GmbZIP44* and *GmbZIP62*, the bZIP genes of soybean (*Glycine max*), conferred tolerance to salt and freezing stress in transgenic *Arabidopsis* plants (Liao et al., 2008). Overexpression of *CabZIP25*, a member of bZIP in pepper (*Capsicum annuum*), enhanced salt tolerance in transgenic *Arabidopsis* and promoted salt sensitivity by decreasing virus induced gene silencing (VIGS) expression in pepper (Gai et al., 2020). The study by Hsieh et al. (2010) showed that *SlAREB*, a member of bZIP in tomato (*Solanum lycopersicum*), regulated stress-responsive genes and improved water logging deficit and salt stress response. Elsewhere, it was reported that *AREB1*, an *Arabidopsis* bZIP transcription factor, conferred tolerance to water deficit (including drought and flooding stresses) in modified soybeans overexpressing *AREB1* (Fuhrmann-Aoyagi et al., 2021). PPI_1_, a bZIP in pepper, regulated expression of genes involved in defense mechanisms (Lee et al., 2002).

The application of genome sequencing has led to identification of bZIP family members (Jakoby et al., 2002), in *Arabidopsis* (Jakoby et al., 2002), rice (E et al., 2014), *Carthamus tinctorius* (Li H. et al., 2020), Chinese jujube (*Ziziphus jujuba*) (Zhang Q. et al., 2020), Olive (*Olea europaea*) (Rong et al., 2020), common bean (*Phaseolus vulgaris*) (Zhang et al., 2021), and potato (*Solanum tuberosum*) (Herath and Verchot, 2020). However, few studies have investigated bZIP family members in adzuki bean (*Vigna angularis*). Adzuki bean (*Vigna angularis*) is an important dietary legume crop that was first cultivated in China (Han et al., 2005). Its grains have high protein content, a low-fat content, and high iron content. They contain several bioactive substances such as triterpenoids, flavonoids, and saponins. It was traditionally used as an iron supplement, to remove damp and swelling (Amarowicz et al., 2008; Yang et al., 2015). Being a sensitive species, adzuki beans are particularly vulnerable to environmental stressors such as cold, drought, salt, and heavy metals (Srivastava et al., 2018; Li W.-Y. et al., 2020). In this study, bZIP members in the adzuki bean were identified, and characterized in terms of phylogeny and evolutionary expansion in different tissues under different stress conditions such as drought (D), cold (C), salt (NaCl) and heavy metal (CdCl_2_). The findings will provide new insights about bZIP members which can be applied in resistance breeding.

## Materials and Methods

### Identification of bZIP Members in *Vigna Angularis*

The basic information for the reference genome (including genes, cDNAs, and proteins) in adzuki bean (Vigan1.1) was obtained from the Esembl plant’s database (https://plants.ensembl.org/Vigna angularis/Info/Index). The bZIP domain information was obtained from the PFAM database (http://pfam.xfam.org/), with PF00170 as the search key. The bZIP members in adzuki bean (*Vigna angularis*) were identified using the HMMER software (Finn et al., 2015) and screened using a database that included the ExPASy Proteomics Server (http://www.expasy.org) (Hoogland et al., 2008) and P3DB (http://www.p3db.org) (Yao and Xu, 2017). After deduplication, the remaining bZIP members were considered to be members of the bZIP family in adzuki bean, and were named VabZIP. VabZIPs were named according to their location in the reference genome in the Esembl database, which was determined using the TBtools software (Chen et al., 2020).

### Analysis of VabZIP Members

Protein sequences of the VabZIP members were aligned using MEGA X (Kumar et al., 2018) while bootstrap values (1,000 replicates) were used for the maximum likelihood analysis. MEGA predicted the optimal model. Ten motifs from VabZIP members were identified using the MEME tool (Bailey et al., 2009), with an E-value of less than 1e^−20^ for motifs containing 10–50 amino acids. Gene structures for VabZIP members were analyzed using GSDS (Hu et al., 2015) and Gene-wise (Simmons et al., 2019), in which the coordinates corresponded to DNA and protein sequences. *Cis*-acting elements of VabZIP members were identified and their functions predicted by the plantCARE software (Lescot et al., 2002). Gene duplication events for VabZIP members were evaluated by MCScanX (Wang et al., 2013) and circus (Krzywinski et al., 2009) software. Subcellular locations for VabZIP members were predicted by the CELLO database (http://cello.life.nctu.edu.tw/) (Yu et al., 2006). Expression data for orthologous genes of VabZIP members in *Arabidopsis* and soybean (*Glycine max*) were obtained from the phytozome database (https://phytozome-next.jgi.doe.gov/).

### Plant Materials, Stress Concentrations, and qRT-PCR Analysis

Plant materials for this study were “Longxiaodou 5”, which was provided by the Institute of Crop Resources, Heilongjiang Academy of Agricultural Sciences (Harbin, Heilongjiang, China). For the seedlings to bud, they were incubated at 26 °C without light (Qi et al., 2021).

During treatment, the following stressors were prioritized: drought, salt, cold, and heavy metals. Salt stress concentration was 70 mmol/L (Zhang Y. et al., 2020) while heavy metal stress concentration was 0.5 mg/L CdCl_2_ (Zhao et al., 2020). A temperature of 4 °C was used to induce cold stress (Wang et al., 2020) while 15% PEG was the concentrate drought stress (Ahmad et al., 2020). The stresses were separately induced on the third day, with water treatment used as the control (CK). The hypocotyl, radicle, cotyledon, and germs were collected as samples for tissue-specific analysis expressions. The radicles were collected in response to these abiotic stress treatments. The RNA Easy Fast Kit (DP452, Tiangen, Beijing) was used for sample RNA extraction, which was used for cDNA synthesis using HiScript SuperMix (R223-01, Vazyme, Nanjing). The *VabZIPs* primers were designed using the Primer premier5.0 software (PREMIER Biosoft, San Francisco, United States ) while *Va-actin* was used as the reference control gene (Li W.-Y. et al., 2020). qRT-PCR analyses for expressions of three biological replicates of each VabZIP member were performed using the Light Cycler system (Roche 480II, Roche, Switzerland) and *TransStart* ^®^ Top Green qPCR SuperMix (AQ131-04, TransGen Biotech, Beijing). Relative expressions were calculated as described by Livak and Schmittgen (2001).

### Subcellular Localization

The coding sequence (CDS) of VabZIP members (VabZIP17 and VabZIP56) was PCR amplified from the cDNAs, which without stop codon. The primers used for cloning the VabZIP17 and VabZIP56 was shown in Supplementary Table S2. Then, the sequence was cloned into the vector, which had the green fluorescent protein (GFP) tag and a CaMV35S promoter. The VabZIP17-GFP and VabZIP56-GFP construct were transformed into Agrobacterium competent cells and transiently expressed in the leaves of Nicotiana benthamiana with the empty vector was used as a control. After injection for 2 days, the leaves were observed under a confocal laser microscope (TCS-SP8 Leica, Wetzlar, Germany) to find fluorescence signals (A1Si, Nikon, Japan).

## Results

### Identification of bZIP Members in *Vigna angularis*

**FIGURE 1:**
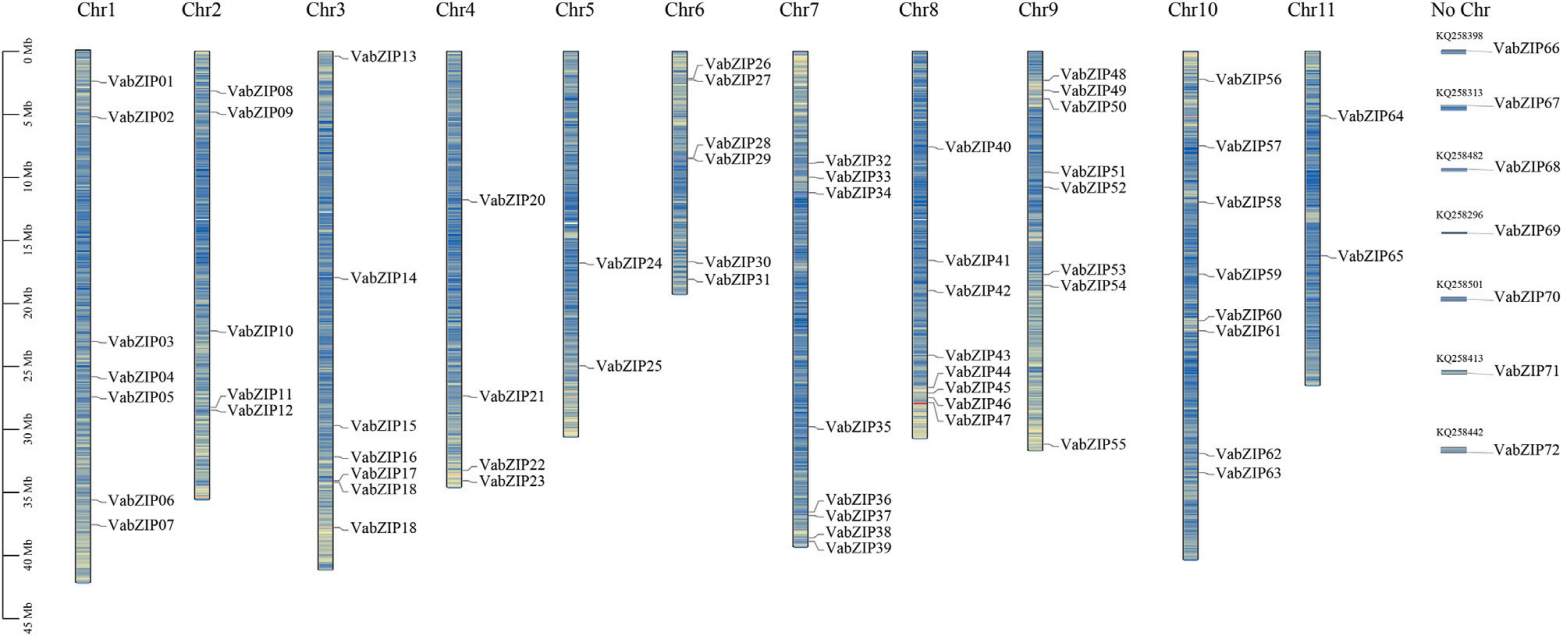
Locations of VabZIP members. The 11 pillars correspond to the 11 chromosomes, whereas No Chr depict members lacking a chromosome. The blue lines indicate gene density on chromosomes.

Following a HMMER-search of the bZIP domain, 72 members of the bZIP family were identified from the reference genome in the Esembl database (*Vigna angularis*), which had no duplications. These members were located on all *Vigna angularis* chromosomes*.* Eight of the members were located on chromosomes 7, 8, 9, and 10 while chromosome 5 had the fewest members (2). Seven members were located on an unknown chromosome, which may be positioned on the scaffold. The bZIP members were named based on their location (VabZIP1-VabZIP72) (Figure 1). Information on the VabZIP members is presented in Supplementary Table S1. Protein lengths of VabZIPs ranged from 80 to 773, with VabZIP56 having the longest protein (773) and CDS (2,322). Isoelectric points of VabZIP members ranged from 4.76–11.56, while their molecular weights ranged from 9,438.77 Kilodalton (Kd) to 84105.11 (Kd) (Supplementary Table S3).

### Evolutionary Analysis of the VabZIP Members

**FIGURE 2:**
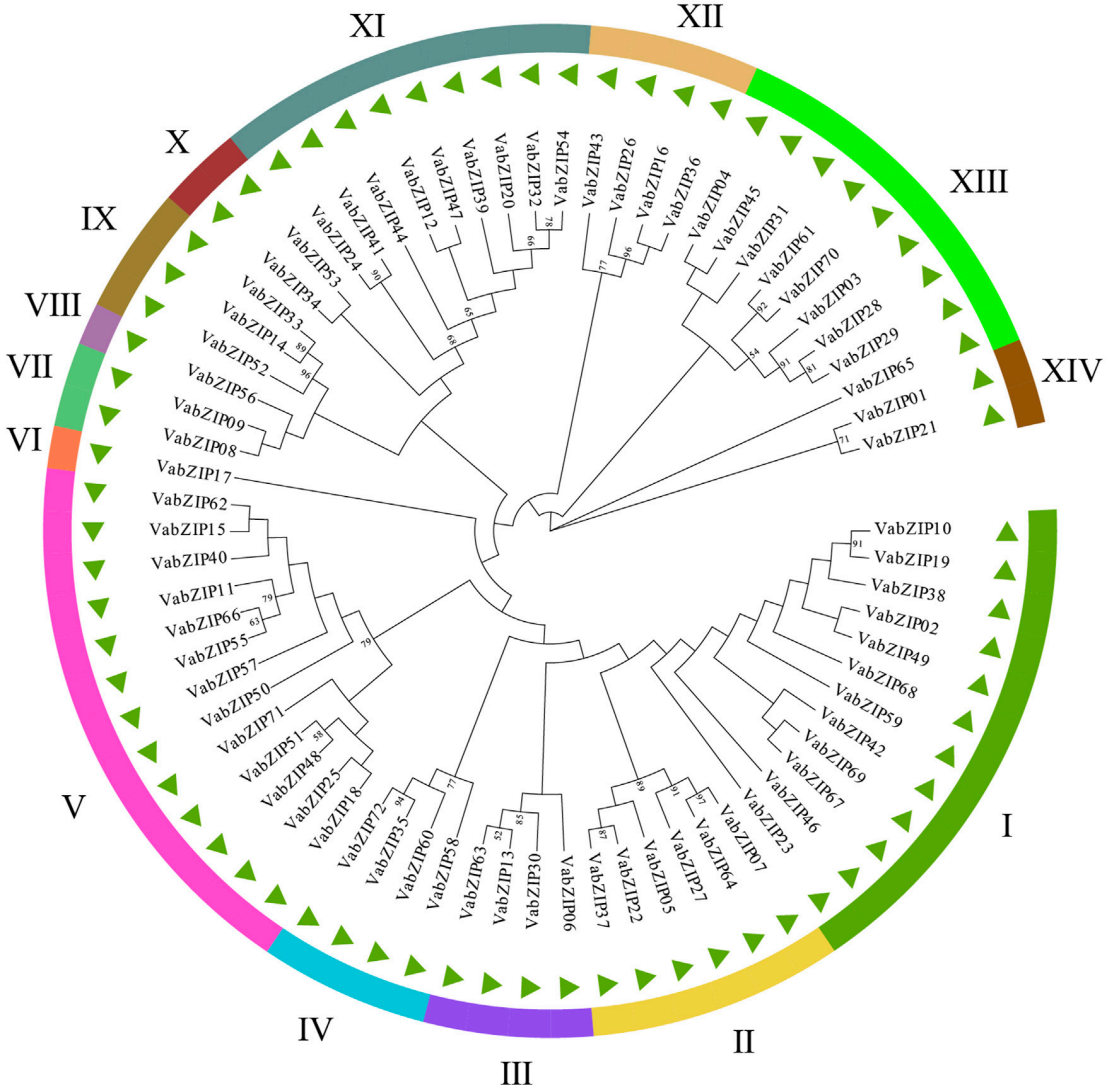
Evolutionary analysis of VabZIPs. Different sub-families are painted in different colors.

Evolutionary history of VabZIP members was determined using the Maximum Likelihoodphy (ML) analysis, with the lg + g model predicted by MEGA X software used as the model for analysis. Findings from MEGA X analysis are presented in Figure 2. These 72 members were divided into 14 subfamilies, with sub-family V having the most VabZIP members (13), while sub-families VI and VIII each had only one VabZIP member, making them the least-membered sub-families. There were 4, 2, and 9 VabZIP members in subfamily III, IV, and XII, subfamily VII, X, and XIV, and subfamily XI and XIII, respectively.

### Motifs and Structure in VabZIPs

**FIGURE 3:**
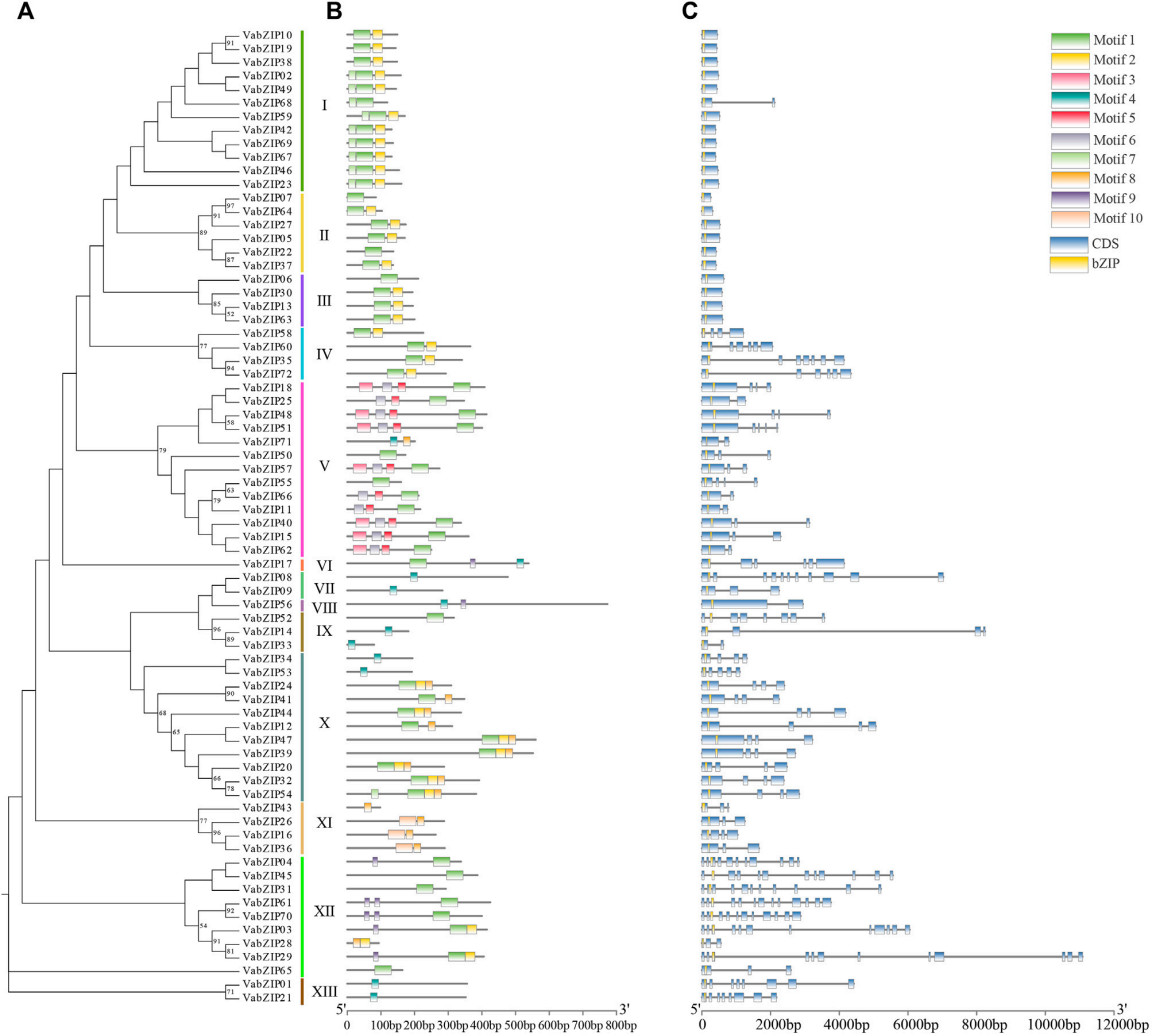
Motifs and gene structure of VabZIPs. **(A)** Evolutionary analysis of VabZIPs. Different colors represent different subfamilies **(B)** The motifs of VabZIPs **(C)** Gene structure of VabZIPs. Blue squares represent CDS while yellow squares represent the bZIP structure.

Analysis of VabZIPs using the MEME software revealed ten motifs (Figures 3A,B and Supplementary Figure S1). Apart from subfamilies VII and VIII, motif-1 was found in most VabZIPs sub-families while motif-3 and motif-5 were only found in sub-family X. Motif 9 was found only in subfamily XII. The VabZIP members in each subfamily had similar motifs. Gene structures for VabZIP members were assessed by GSDS, which revealed exon and intron structures (Figure 3C). The bZIP structure was located above all members in these VabZIPs, and sub-families I to III members had shorter introns than members of the other subfamilies.

### Evolution of bZIP Members in Two Species

**FIGURE 4:**
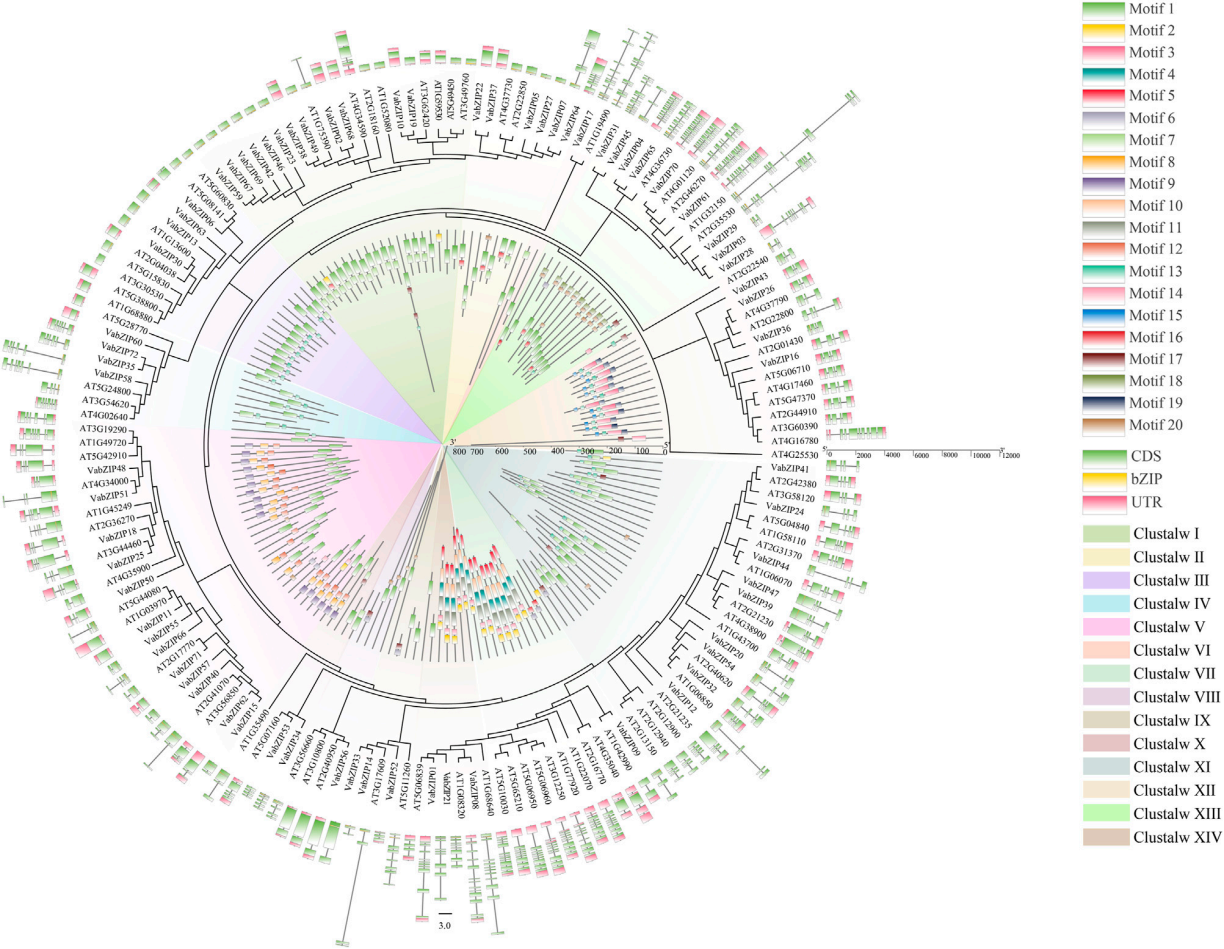
Evolution of bZIP members in *Arabidopsis* and VabZIPs. The inner ring indicates the motifs of bZIP members while the outer ring is the gene structure of bZIP members.

The 72 VabZIPs were compared to sequences encoded by bZIP members from *Arabidopsis* to determine their evolutionary history, motif, and gene structure (Figure 4 and Supplementary Figure S2). MEGA X predicted the lg + g model as the best model, and 14 subfamilies were defined based on the results in these two species, consistent with the evolutionary of VabZIPs (Figure 2). Each subfamily had bZIP members of these two species. Each member of the subfamily had comparable motifs and gene structures.

### *Cis*-Elements of *VabZIPs*

**FIGURE 5:**
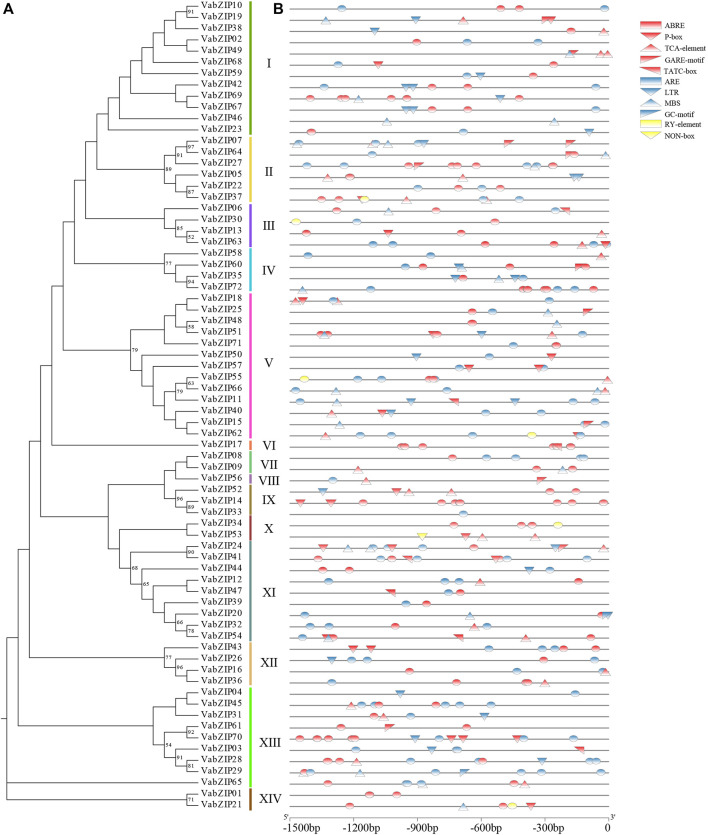
*Cis*-elements of *VabZIPs.* **(A)** Evolutionary analysis of *VabZIPs.* **(B)** *Cis*-elements of *VabZIPs.* The red models represent the hormone-related elements. The blue models represent the stress-related elements. The yellow models represent the elements in germination.

*Cis*-elements of *VabZIP* members were analyzed using the plantCARE software. PlantCARE predicted the functions of the ten *cis*-elements (Supplementary Table S4), which were divided into three categories: hormone responsiveness (red), environmental stress (blue), and germination (yellow). Hormone responsiveness elements, including TATC-box, P-box, and GARE-motif were involved in gibberellin responsiveness, while ABRE was the *cis*-acting element involved in abscisic acid responsiveness. Environmental stress elements, such as LTR were involved in low-temperature responsiveness, while MBS was involved in drought-inducibility. The RY-element and NON-box elements had seed-specific regulation function. These findings indicate that *VabZIP* family members are involved in hormone regulation, stress resistance, and seed germination (Figure 5).

### Collinearity Analysis of *VabZIPs*

**FIGURE 6:**
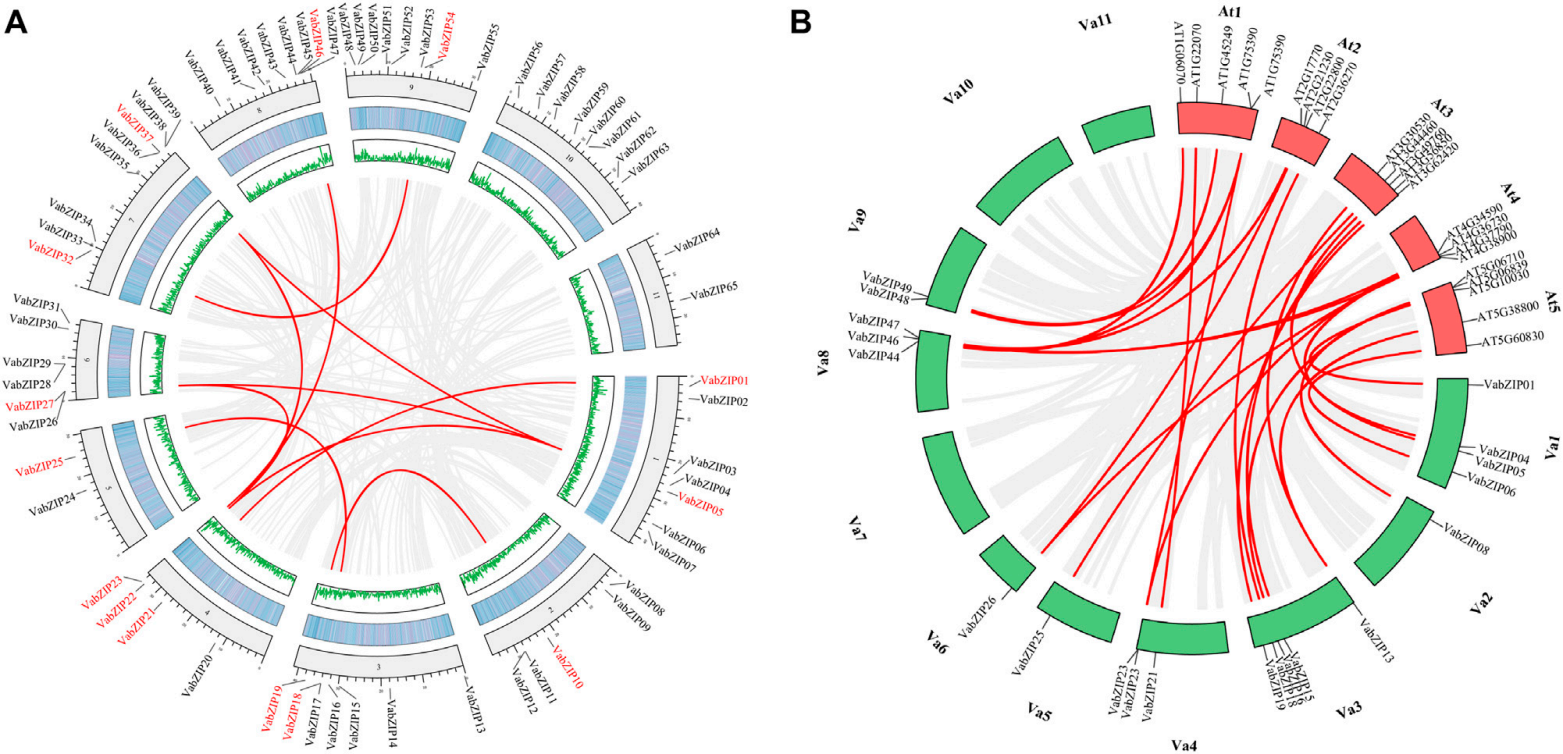
Collinearity analysis of *VabZIPs.* **(A)** Collinearity of *PvHsf* members. The red *PvHsfs* indicate collinearity while the black ones have no collinearity. The middle two rings indicate gene density. The gray background line indicates a collinear background while the green lines indicate a collinear relationship. **(B)** Collinearity of *VabZIPs* with *Arabidopsis*. Red boxes are the chromosomes of *Arabidopsis* while the green boxes are the chromosomes of the adzuki bean. The gray lines indicate the collinearity background while the red lines indicate collinearity between *VabZIPs* and *Arabidopsis* members.

There was collinearity between ten pairs of *VabZIPs* with *VABZIP05* and *VABZIP22* being the most collinear with other *VabZIP* members (three pairs). *VaBZIP27* and *VaBZIP37* had two pairs of collinearity (Figure 6A). Twenty five collinearity pairs were identified between *VabZIP* members and *Arabidopsis*, with *VabZIP13*, *VabZIP23*, *VaBZIP26*, *VaBZIP46,* and *VaBZIP47* having two collinear members in *Arabidopsis*, implying that the 21 *VabZIP* members may have the same function as collinear *Arabidopsis genes* (Figure 6B)*.*

### Subcellular Location Analysis of VabZIPs

Subcellular locations for VabZIPs were analyzed using the CELLO database, with locations predicted by Molecular bioinformatics center. Almost all VabZIP members were predicted to be expressed in the nucleus, with only VabZIP11 predicted to be located on chloroplasts or in the cytoplasm (Supplementary Table S5).

**FIGURE 7:**
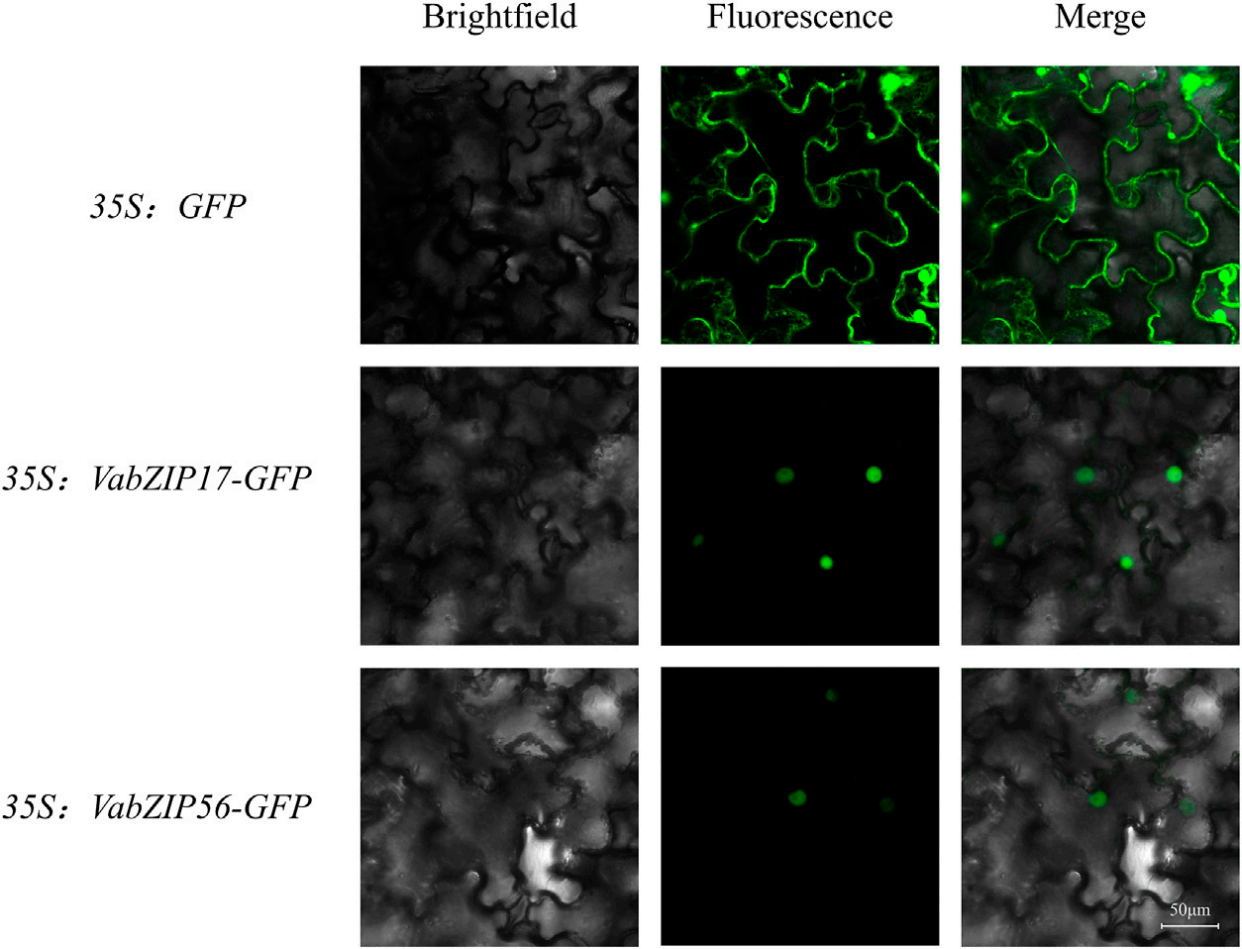
The subcellular localization of VabZIP17 and VabZIP56.

In order to analyze the subcellular localization of VabZIP members, two members in different subfamilies were selected (VabZIP17 and VabZIP56) for subcellular location analysis. The result showed that the control (GFP) was distributed on the membrane and nuclear, while the VabZIP17-GFP and VabZIP56-GFP fusion proteins were only found on the nuclear, which indicated VabZIP17 and VabZIP56 were located on the nuclear (Figure 7).

### Tissue-specific Expression Analysis at the Bud Stage

**FIGURE 8:**
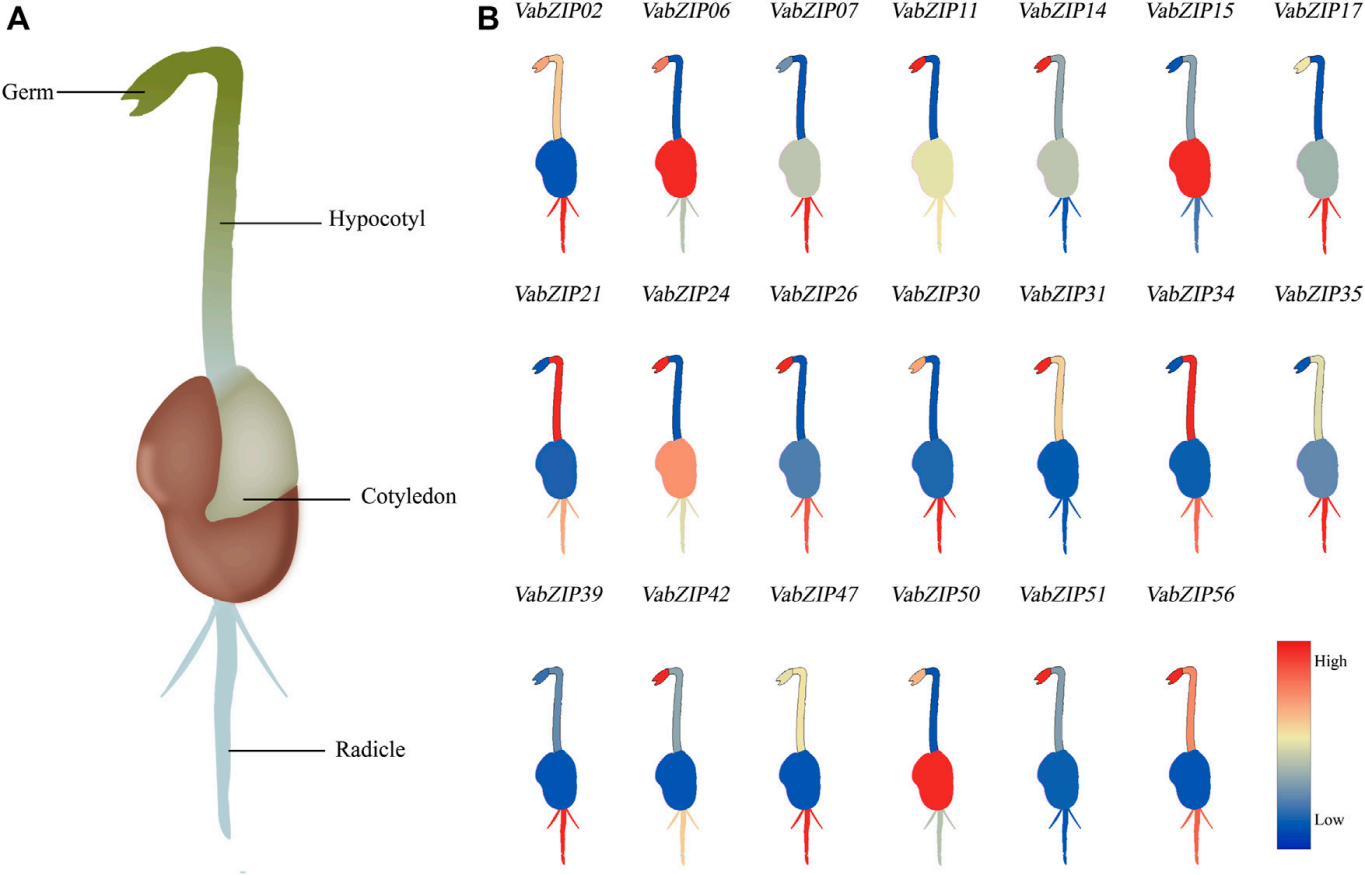
Tissue-specific expression analysis of *VabZIPs* at the bud stage. **(A)** Schematic illustration of tissues at the bud stage of adzuki bean. **(B)** Expressions of *VabZIPs* in different tissues. The change in color from red to blue indicates a high to low expression.

During the budding stage, the germ, cotyledon, hypocotyl, and radicle were used to investigate the expressions of bZIP members in different tissues in the adzuki bean. The twenty *VabZIP* members were selected randomly for qRT-PCR analysis which these twenty *VabZIP* members covered all of the sub-families. The *VabZIP* members were found to be expressed in a tissue-specific manner. *VabZIP06* was abundantly expressed in the germ and cotyledon, while *VabZIP11* and *VabZIP26* were highly expressed in the germ. The radicle was highly enriched with *VabZIP17*, *VabZIP30*, *VabZIP35,* and *VabZIP47,* compared to the other tissues (Figure 8).

### Expressions of *VabZIPs* Under Abiotic Stress at the Budding Stage

**FIGURE 9:**
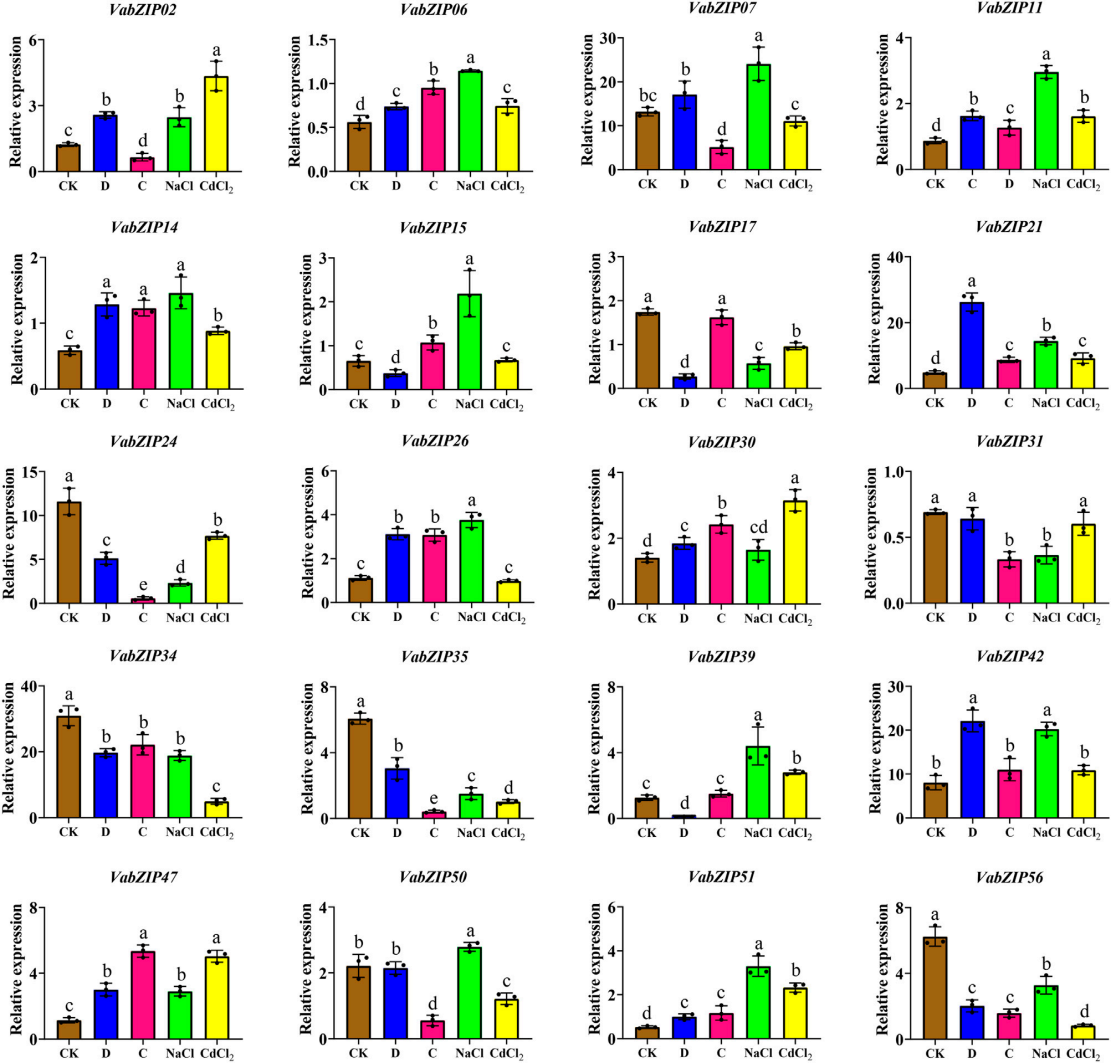
Relative expressions of *VabZIPs* in radicles under different abiotic stress levels at the bud stage. Brown squares denote CK treatment, whereas the blue, pink, green, and yellow squares denote drought, cold, salt, and heavy metal stress, respectively.

Also, these twenty *VabZIP* members were selected randomly for qRT-PCR analysis to determine variations in expressions in response to abiotic stress. Expressions of the nine *VabZIP* members varied in response to various stresses (Figure 9). Expressions of some *VabZIP* members (such as *VabZIP06*, *VabZIP11*, *VabZIP21*, *VabZIP47* and *VabZIP51*) were up-regulated in response to drought, cold, salt, and heavy metal stress, whereas others *VabZIP* members (such as *VabZIP24*, *VabZIP34*, *VabZIP35* and *VabZIP56*) were down-regulated. Differences in expressions of *VabZIPs* in response to various types of abiotic stress were significant, such that while *VabZIP26* and *VabZIP15* did not exhibit marked changes in response to heavy metal stress, they did change significantly in response to drought and cold stress, indicating that these two members may respond to other abiotic stressors other than heavy metals.

## Discussion

The bZIP members are present in various species, and the number of members vary from one species to another. There are 78 members in *Arabidopsis* (Dröge-Laser et al., 2018), 80 members in potato (*Solanum tuberosum*) (Herath and Verchot, 2020), 160 members in soybean (Glycine max) (Zhang et al., 2018), 89 members in rice (*Oryza sativa* L.) (Nijhawan et al., 2008) and 69 members in tomato (*Solanum lycopersicum*) (Li X. et al., 2015). In this study, VabZIP members were identified from the reference genome of the adzuki bean, which contained 72 members. The number of VabZIP members was found to exceed the number of bZIP members in tomato, while the number of bZIP members was less than in *Arabidopsis*, potato, soybean, and rice. These findings suggest that the number of bZIP members may be related to the size of the reference genome and that after differentiation from their early ancestors, the adzuki bean may have experienced fewer genomic replication events, when compared to other species.

Evolutionary analysis revealed that VabZIP members could be divided into 14 subfamilies in the unrooted Maximum Likelihoodphy (ML) tree, and the result of evolutionary combined with bZIP members in *Arabidopsis* and adzuki bean was also revealed that bZIP members had 14 sub-families, which was similar to the results in wheat (*Triticum aestivum*) (Li D. et al., 2015) and Chinese jujube (*Ziziphus jujuba* Mill.) (Zhang Q. et al., 2020). With regards to motif and gene structures of VabZIP members, motif constitutions differed in different sub-families. Within the same sub-family, the motifs were similar and the motif of the coded bZIP (motif-1) was highly conserved (Zhou et al., 2017). Motif-3 and motif-5 were only found in sub-family X while motif 9 was only found in subfamily XII, which was similarly found in tartaty buckwheat (*Fagopyrum tataricum*) (Liu et al., 2019). VabZIP members from the same subfamily exhibited a similar gene structure, whereas VabZIPs from sub-families I, II and, III had no more than two introns, suggesting a relationship between the low number of introns and stress responses in the three sub-families (Zhao et al., 2016). The combined results of the motif and gene structure for two species of bZIP members revealed similar results.

*Cis*-elements in promoter regions of *VabZIP* members regulate the functions of *VabZIPs* that contain related *cis*-elements (Lescot et al., 2002). Similarly, bZIP members have *cis*-elements that are comparable across species: ABRE, TATC-box, TCA-element, and P-box are hormone-related *cis*-elements in *VabZIP* and bZIP members in common bean and potato (Wang et al., 2021; Zhang et al., 2021), implying that *VabZIPs* may regulate hormones involved in plant growth. Stress-related *cis*-elements, such as MBS and LTR, were found in *VabZIPs* and sesame bZIP transcription factor members (Wang et al., 2018), leading to the hypothesis that *VabZIPs* are involved in abiotic stress responses. Moreover, since they contain the RY element, which is similar to that found in the common bean, *VabZIP* members may have had an effect at the bud stage (Zhang et al., 2021).

Collinear analysis allows the transfer of functional information from a well-studied taxon to a less-studied taxon (Ghiurcuta and Moret, 2014). In this study, 25 *VabZIPs* pairs exhibited collinearity with an *Arabidopsis* member, which was found to be involved in plant growth regulation, abiotic stress responses, responses to hormones, and germination in plants. *AT4G37790*, *AT1G45249,* and *AT2G36270* were the collinearity genes for *VABZIP26*, *VaBZIP48,* and *VaBZIP18* respectively, which play a function in salt stress responses (Lopez-Molina et al., 2001; Nakashima et al., 2006; Liu et al., 2016). Collinearity members for *Vigna angularis* such as *VaBZIP23*, *VaBZIP46*, *VaBZIP18,* and *VaBZIP47* in *Arabidopsis*, have a role in the bud stage of the plant: *AT1G75390*, the collinearity member for *VabZIP23* and *VabZIP46*, positively regulates plant seed germination rate. Its knock-out was associated with significantly slower germination rate (Iglesias-Fernández et al., 2013). *AT2G36270* had collinearity with *VabZIP18*, which increases proteins for preventing seed germination (Piskurewicz et al., 2008); *AT4G38900*, the collinearity gene for *VabZIP47* was expressed in meristematic tissues and negatively modulates *Arabidopsis* growth (Lozano-Sotomayor et al., 2016). In *Arabidopsis*, *VaBZIPs* member collinearity genes, such as *AT1G22070* (*VaBZIP21*) were shown to be involved in the salicylic acid (SA) signaling pathway (Zhou et al., 2000), whereas *AT1G45249* (*VaBZIP48*) and *AT2G36270* (*VaBZIP18*) were found to be involved in abscisic acid (ABA) responses (Fujita et al., 2005). The collinear analysis results indicate that *VaBZIP* members are involved in responses to hormones, coping with environmental pressures, and regulating the bud stage.

The bZIP members have previously been reported to exhibit tissue-specific expressions, including in Olive (*Olea europaea*) (Rong et al., 2020), radish (*Raphanus sativus*) (Fan et al., 2019), and poplar (Zhao et al., 2021). Expressions of *VabZIPs* at the bud stage revealed tissue-specificity, with the radicle having higher expressions than other tissues, indicating that the radicle could be used as a target tissue for *VabZIPs’* research. Gene expression changes under abiotic stress conditions might lead to abiotic stress responses in plants, and differentially expressed genes under abiotic stress can be used as candidate genes for further research on abiotic stress responses (Qi et al., 2021). In this study, expressions of selected *VabZIPs* under abiotic stress indicated that *VabZIPs* are involved in abiotic stress responses. Expressions of *VabZIP06*, *VabZIP11*, *VabZIP21*, *VabZIP47* and *VabZIP51* were markedly up-regulates under drought, cold, salt and heavy metal stress, implying that these bZIP members are involved in abiotic stress responses. Moreover, some bZIP members are involved in abiotic stress responses in other plants, such as *StbZIP25* in potato (Wang et al., 2021), *TabZIP96* in wheat (Liang et al., 2022) and *CabZIP25* in pepper (Gai et al., 2020). Expressions of bZIP members under abiotic stress revealed that some *bZIPs* can be used in plant breeding for abiotic stress resistance, such as in watermelon (Yang et al., 2019), sesame (Wang et al., 2018), and apple (Zhao et al., 2016). These results indicate that *bZIPs* might be useful in molecular breeding under abiotic stress and the *VabZIPs* that were differentially expressed under stress can be used for further research, particularly in stress-resistance breeding.

## Conclusion

In this study, 72 VabZIP members were identified and divided into 14 subfamilies. The members of each sub-family had motifs and gene structures that were comparable. These VabZIP members exhibited hormonal responsiveness, environmental stress, and germination *cis*-elements, indicating that the *VabZIPs* might be involved in plant hormone and abiotic stress regulation. The *VabZIPs,* whose expressions were tissue specific, might be involved in abiotic stress responses. And VabZIP17 and VabZIP56 were located on the nuclear in subcellular localization ananlysis. Furthermore, expressions of *VabZIPs* under stress conditions such as drought, cold, salt, and heavy metal stress at the bud stage revealed that some *VabZIPs* (such as *VabZIP06*, *VabZIP11*, *VabZIP21*, *VabZIP47* and *VabZIP51*) might regulate abiotic stresses responses in the adzuki bean. This study provides valuable information and insights into the development of *VabZIPs* and establishes a foundation for the use of related characteristics of *VabZIPs* in adzuki beans.

## Data Availability

The datasets presented in this study can be found in online repositories. The names of the repository/repositories and accession number(s) can be found in the article/Supplementary Material.
