# Supplementary material for: The bZIP Transcription Factor Family in Adzuki Bean (Vigna Angularis): Genome-Wide Identification, Evolution, and Expression Under Abiotic Stress During the Bud Stage
Source: Front Genet. 2022 Apr 25;13:847612. doi: 10.3389/fgene.2022.847612 (PMC9081612; doi:10.3389/fgene.2022.847612)
Supplement: Supplementary file 1 [file Table1.DOCX]

Table S1: QRT-PCR primers for *VabZIPs.*

| Primer name | Primer sequence (5’-3’) |
| --- | --- |
| *VaActinF* | CTAAGGCTAATCGTGAGAA |
| *VaActinR* | CGTAAATAGGAACCGTGT |
| *VabZIP02F* | CTCTTCAAAACTCTGGCTC |
| *VabZIP02R* | TCATTTCATTGTTCTCCTT |
| *VabZIP06F* | GTAGCCCCTTATGCTTCCTT |
| *VabZIP06R* | GTTGGTCCAGATGGGTCA |
| *VabZIP07F* | AAACGCACGAGAATGATAT |
| *VabZIP07R* | ACAATGGTGAACGAGAAAC |
| *VabZIP11F* | TTACCACCACCAACTTCATC |
| *VabZIP11R* | AGTCATCATCTCGTCGTCAG |
| *VabZIP14F* | TTCACTGTTCCAGATGTGG |
| *VabZIP14R* | GTTTGTGGCGTCTATGCTC |
| *VabZIP15F* | GACTCTGGAGGATTTCTTGG |
| *VabZIP15R* | GTTGTGATGTGTTGTGTTGG |
| *VabZIP17F* | GGCACAAATCGCCAAGTCAA |
| *VabZIP17R* | AAGTAGGCTGAAGGGGCGAA |
| *VabZIP21F* | *GTATTAAAAATCTCCGCCA* |
| *VabZIP21R* | *CGAAGTTTCCTACACCCAA* |
| *VabZIP24F* | *GCTGATTTCCATGTTCTCT* |
| *VabZIP24R* | *TCTCCTCTTCAACCTTTTC* |
| *VabZIP26F* | GCCAAGCAAGTGCCCAA |
| *VabZIP26R* | CTCTTCACAACCCTCCCAGT |
| *VabZIP30F* | ACTTTCTTGCTCCTCCCTCC |
| *VabZIP30R* | TGCTTTTGCTTCCTCATCCT |
| *VabZIP31F* | ACAAGGATAGAGTGTTGTG |
| *VabZIP31R* | TAGTTCAGTGTTAGTGCCA |
| *VabZIP34F* | AGAGGGAACCCAGTAGAAG |
| *VabZIP34R* | TCTGGAATGGTGGCAAG |
| *VabZIP35F* | GCTTCTGGTCACCTCAACGA |
| *VabZIP35R* | TTTTCCGAGTAGAAGACCGT |
| *VabZIP39F* | TTCACCTTCTTCAGCCTATC |
| *VabZIP39R* | AGAGAGAAAAAAGAGGGCTG |
| *VabZIP42F* | CAGCAGATACTCACCACCG |
| *VabZIP42R* | TTCCCCTCTCCTTCTCACA |
| *VabZIP47F* | TACATATCAGAGCTAGAGCA |
| *VabZIP47R* | TTAGTAAGTCCAGCAGAATC |
| *VabZIP50F* | CGCCACCAAGATCAACACC |
| *VabZIP50R* | CTTCCGTCAACCGAGCAAT |
| *VabZIP51F* | ATGTTGGACTGGTTGGTTT |
| *VabZIP51R* | TTTCTTCCCCTGTTGATTG |
| *VabZIP56F* | ATGTGACCCCAATAATACC |
| *VabZIP56R* | TTGAAAAACCTTGAAACGC |
