# Supplementary figures and images for: The bZIP Transcription Factor Family in Adzuki Bean (Vigna Angularis): Genome-Wide Identification, Evolution, and Expression Under Abiotic Stress During the Bud Stage
Source: Front Genet. 2022 Apr 25;13:847612. doi: 10.3389/fgene.2022.847612 (PMC9081612; doi:10.3389/fgene.2022.847612)

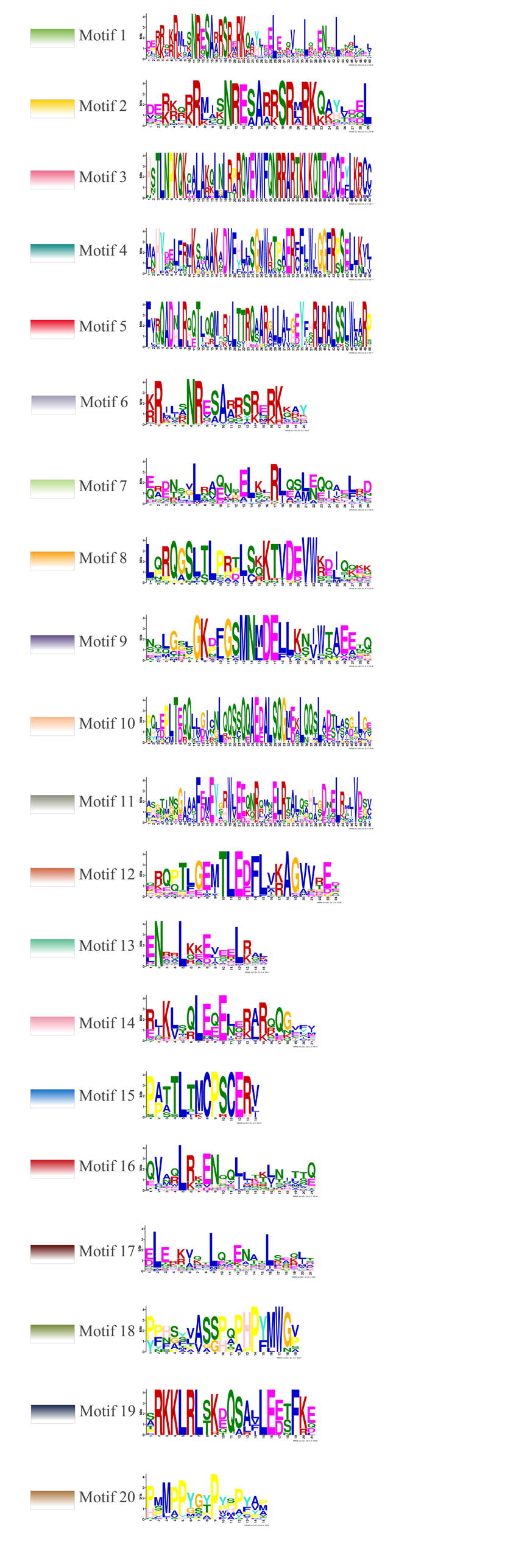

Supplement: Supplementary file 3 [file Image2.JPEG]
