# Supplementary material for: The bZIP Transcription Factor Family in Adzuki Bean (Vigna Angularis): Genome-Wide Identification, Evolution, and Expression Under Abiotic Stress During the Bud Stage
Source: Front Genet. 2022 Apr 25;13:847612. doi: 10.3389/fgene.2022.847612 (PMC9081612; doi:10.3389/fgene.2022.847612)
Supplement: Supplementary file 4 [file Table2.DOCX]

Table S2: The primers of cloning the VabZIP17 and VabZIP56.

| Primer name | Primer sequence (5’-3’) |
| --- | --- |
| *CVabZIP17F* | attgattagagatcttctagaATGGATGCTGAATCGGAGAAGC |
| *CVabZIP17R* | aatgtcgacggtaccggatccACACTGCATTCGAGACTGGCG |
| *CVabZIP56F* | attgattagagatcttctagaATGACCGAATCAATGCACGC |
| *CVabZIP56R* | aatgtcgacggtaccggatccCCAAAGGGGAGAAGCACGA |
