# Supplementary material for: The bZIP Transcription Factor Family in Adzuki Bean (Vigna Angularis): Genome-Wide Identification, Evolution, and Expression Under Abiotic Stress During the Bud Stage
Source: Front Genet. 2022 Apr 25;13:847612. doi: 10.3389/fgene.2022.847612 (PMC9081612; doi:10.3389/fgene.2022.847612)
Supplement: Supplementary file 5 [file Table5.DOCX]

Table S5: Predicted subcellular locations of VabZIPs.

| Number | Subcellular location | Number | Subcellular location |
| --- | --- | --- | --- |
| VabZIP01 | Nuclear | VabZIP37 | Nuclear |
| VabZIP02 | Nuclear | VabZIP38 | Nuclear |
| VabZIP03 | Nuclear | VabZIP39 | Nuclear |
| VabZIP04 | Nuclear | VabZIP40 | Nuclear |
| VabZIP05 | Nuclear | VabZIP41 | Nuclear |
| VabZIP06 | Nuclear | VabZIP42 | Nuclear |
| VabZIP07 | Nuclear | VabZIP43 | Nuclear |
| VabZIP08 | Nuclear | VabZIP44 | Nuclear |
| VabZIP09 | Nuclear | VabZIP45 | Nuclear |
| VabZIP10 | Nuclear | VabZIP46 | Nuclear |
| VabZIP11 | Chloroplast/ Cytoplasmic | VabZIP47 | Nuclear |
| VabZIP12 | Nuclear | VabZIP48 | Nuclear |
| VabZIP13 | Nuclear | VabZIP49 | Nuclear |
| VabZIP14 | Nuclear | VabZIP50 | Nuclear |
| VabZIP15 | Nuclear | VabZIP51 | Nuclear |
| VabZIP16 | Nuclear | VabZIP52 | Nuclear |
| VabZIP17 | Nuclear | VabZIP53 | Nuclear |
| VabZIP18 | Nuclear | VabZIP54 | Nuclear |
| VabZIP19 | Nuclear | VabZIP55 | Nuclear |
| VabZIP20 | Nuclear | VabZIP56 | Nuclear |
| VabZIP21 | Nuclear | VabZIP57 | Nuclear |
| VabZIP22 | Nuclear | VabZIP58 | Nuclear |
| VabZIP23 | Nuclear | VabZIP59 | Nuclear |
| VabZIP24 | Nuclear | VabZIP60 | Nuclear |
| VabZIP25 | Nuclear | VabZIP61 | Nuclear |
| VabZIP26 | Nuclear | VabZIP62 | Nuclear |
| VabZIP27 | Nuclear | VabZIP63 | Nuclear |
| VabZIP28 | Nuclear | VabZIP64 | Nuclear |
| VabZIP29 | Nuclear | VabZIP65 | Nuclear |
| VabZIP30 | Nuclear | VabZIP66 | Nuclear |
| VabZIP31 | Nuclear | VabZIP67 | Nuclear |
| VabZIP32 | Nuclear | VabZIP68 | Nuclear |
| VabZIP33 | Nuclear | VabZIP69 | Nuclear |
| VabZIP34 | Nuclear | VabZIP70 | Nuclear |
| VabZIP35 | Nuclear | VabZIP71 | Nuclear |
| VabZIP36 | Nuclear | VabZIP72 | Nuclear |
