# Supplementary material for: The bZIP Transcription Factor Family in Adzuki Bean (Vigna Angularis): Genome-Wide Identification, Evolution, and Expression Under Abiotic Stress During the Bud Stage
Source: Front Genet. 2022 Apr 25;13:847612. doi: 10.3389/fgene.2022.847612 (PMC9081612; doi:10.3389/fgene.2022.847612)
Supplement: Supplementary file 6 [file Table3.DOCX]

Table S3: Basic information of VabZIPs.

| Gene name | Gene accession | Gene_id | Chr | Location | Protein length | CDS length | Lsoelectric point | Molecular weith | Instability index | Aliphatic index |
| --- | --- | --- | --- | --- | --- | --- | --- | --- | --- | --- |
| VabZIP01 | KOM30669 | Vigan01g022300 | 1 | 2350207:2354632 | 356 | 1071 | 6.15 | 40827.57 | 45.44 | 86.04 |
| VabZIP02 | KOM30928 | Vigan01g048300 | 1 | 5188727:5189206 | 159 | 480 | 5.25 | 17956.12 | 48.67 | 63.9 |
| VabZIP03 | KOM32090 | Vigan01g164600 | 1 | 23004869:23010921 | 415 | 1248 | 6.03 | 44370.73 | 57.05 | 53.64 |
| VabZIP04 | KOM32314 | Vigan01g187000 | 1 | 25817296:25820124 | 338 | 1017 | 5.61 | 35906.5 | 69.38 | 50.3 |
| VabZIP05 | KOM32440 | Vigan01g199600 | 1 | 27417489:27418004 | 171 | 516 | 9.56 | 19917.61 | 60.84 | 79.18 |
| VabZIP06 | KOM33143 | Vigan01g269900 | 1 | 35599631:35600266 | 211 | 636 | 7.29 | 24088.98 | 60.78 | 71.18 |
| VabZIP07 | KOM33331 | Vigan01g288700 | 1 | 37550920:37551177 | 85 | 258 | 11.56 | 10580.32 | 62.26 | 73.41 |
| VabZIP08 | KOM34126 | Vigan02g027600 | 2 | 3133467:3140497 | 477 | 1434 | 7.76 | 53344.42 | 60.81 | 73.9 |
| VabZIP09 | KOM34252 | Vigan02g040200 | 2 | 4814273:4816520 | 283 | 852 | 5.04 | 32019.38 | 63.63 | 80.92 |
| VabZIP10 | KOM35333 | Vigan02g148300 | 2 | 22171744:22172193 | 149 | 450 | 5.3 | 16849.05 | 55.35 | 77.38 |
| VabZIP11 | KOM35871 | Vigan02g202100 | 2 | 28242137:28242884 | 217 | 654 | 4.87 | 23935.91 | 46.01 | 72.81 |
| VabZIP12 | KOM35892 | Vigan02g204200 | 2 | 28454300:28459364 | 312 | 939 | 6.41 | 34562.37 | 55.81 | 84.71 |
| VabZIP13 | KOM36660 | Vigan03g004100 | 3 | 378777:379364 | 195 | 588 | 5.82 | 22505 | 69.73 | 64.46 |
| VabZIP14 | KOM37905 | Vigan03g128700 | 3 | 17940292:17948538 | 182 | 549 | 8.45 | 20346.51 | 50.1 | 60.49 |
| VabZIP15 | KOM38669 | Vigan03g205100 | 3 | 29680775:29683068 | 361 | 1086 | 7.06 | 40482.69 | 49.73 | 73.74 |
| VabZIP16 | KOM38851 | Vigan03g223300 | 3 | 32166198:32167241 | 263 | 792 | 7.62 | 29249.09 | 48.93 | 72.02 |
| VabZIP17 | KOM39025 | Vigan03g240700 | 3 | 34063657:34067799 | 539 | 1620 | 6.31 | 60290.02 | 64.75 | 62.84 |
| VabZIP18 | KOM39038 | Vigan03g242000 | 3 | 34199563:34201561 | 408 | 1227 | 6.62 | 45183.26 | 58.24 | 60.22 |
| VabZIP19 | KOM39406 | Vigan03g278800 | 3 | 37777935:37778369 | 144 | 435 | 7.87 | 16252.55 | 62.45 | 84.79 |
| VabZIP20 | KOM40664 | Vigan04g086200 | 4 | 11767033:11769509 | 288 | 867 | 5.58 | 32103.78 | 66.41 | 64.79 |
| VabZIP21 | KOM41651 | Vigan04g184900 | 4 | 27332876:27335049 | 352 | 1059 | 6.42 | 39711.35 | 54.69 | 92.84 |
| VabZIP22 | KOM42202 | Vigan04g240000 | 4 | 33247977:33248390 | 137 | 414 | 10.5 | 15848.15 | 66.48 | 84.74 |
| VabZIP23 | KOM42320 | Vigan04g251800 | 4 | 34066058:34066543 | 161 | 486 | 6.42 | 17893.98 | 56.93 | 72.73 |
| VabZIP24 | KOM43516 | Vigan05g112000 | 5 | 16782385:16784786 | 309 | 930 | 6.46 | 34302.32 | 64.27 | 75.57 |
| VabZIP25 | KOM44081 | Vigan05g168600 | 5 | 24937416:24938684 | 347 | 1044 | 4.78 | 39403.04 | 55.62 | 74.44 |
| VabZIP26 | KOM44924 | Vigan06g023000 | 6 | 2141688:2142938 | 288 | 867 | 8.33 | 32310.45 | 60.89 | 63.68 |
| VabZIP27 | KOM44931 | Vigan06g023700 | 6 | 2246192:2246716 | 174 | 525 | 10 | 20348.97 | 64.45 | 67.87 |
| VabZIP28 | KOM45498 | Vigan06g080400 | 6 | 8452741:8453295 | 94 | 285 | 4.76 | 10418.21 | 54.37 | 68.62 |
| VabZIP29 | KOM45504 | Vigan06g081000 | 6 | 8515695:8526781 | 406 | 1221 | 5.9 | 43283.4 | 62.6 | 48.65 |
| VabZIP30 | KOM46058 | Vigan06g136400 | 6 | 16679835:16680419 | 194 | 585 | 5.62 | 22336.09 | 63.33 | 74.9 |
| VabZIP31 | KOM46176 | Vigan06g148200 | 6 | 18071262:18076475 | 293 | 882 | 5.39 | 33624.93 | 58.83 | 73.58 |
| VabZIP32 | KOM47150 | Vigan07g085400 | 7 | 8862379:8864765 | 392 | 1179 | 6.17 | 42468.99 | 55.87 | 62.09 |
| VabZIP33 | KOM47230 | Vigan07g093400 | 7 | 9974139:9974765 | 80 | 243 | 9.89 | 9438.77 | 35.12 | 84.12 |
| VabZIP34 | KOM47344 | Vigan07g104800 | 7 | 11202229:11203539 | 194 | 585 | 9.6 | 22740.07 | 47.07 | 84.48 |
| VabZIP35 | KOM48431 | Vigan07g213500 | 7 | 29774700:29778837 | 341 | 1026 | 5.51 | 37311.29 | 44.75 | 68.09 |
| VabZIP36 | KOM48904 | Vigan07g260800 | 7 | 36539405:36541069 | 290 | 873 | 5.48 | 32895.37 | 61.23 | 60.52 |
| VabZIP37 | KOM48921 | Vigan07g262500 | 7 | 36820998:36821408 | 136 | 411 | 9.83 | 16221.59 | 81.19 | 86.03 |
| VabZIP38 | KOM49096 | Vigan07g280000 | 7 | 38589436:38589882 | 148 | 447 | 9.52 | 17331.62 | 67.5 | 82.43 |
| VabZIP39 | KOM49127 | Vigan07g283100 | 7 | 38864262:38866983 | 552 | 1659 | 6.97 | 60460.58 | 63.96 | 65.74 |
| VabZIP40 | KOM49637 | Vigan08g046400 | 8 | 7559112:7562242 | 338 | 1017 | 6.88 | 37432.43 | 63.94 | 63.46 |
| VabZIP41 | KOM50101 | Vigan08g092800 | 8 | 16566844:16569089 | 348 | 1047 | 5.58 | 39412.4 | 59.05 | 57.21 |
| VabZIP42 | KOM50248 | Vigan08g107500 | 8 | 18969883:18970281 | 132 | 399 | 7.83 | 14958.85 | 67.56 | 82.65 |
| VabZIP43 | KOM50614 | Vigan08g144100 | 8 | 24097379:24098155 | 98 | 297 | 5.28 | 11272.47 | 54.1 | 83.78 |
| VabZIP44 | KOM50818 | Vigan08g164500 | 8 | 26665589:26669773 | 338 | 1017 | 5.99 | 38363.5 | 53.63 | 72.46 |
| VabZIP45 | KOM50875 | Vigan08g170200 | 8 | 27110259:27115820 | 387 | 1164 | 9.35 | 43306.27 | 54.84 | 60.52 |
| VabZIP46 | KOM50919 | Vigan08g174600 | 8 | 27486247:27486711 | 154 | 465 | 6.74 | 17213.4 | 50.54 | 81.69 |
| VabZIP47 | KOM50955 | Vigan08g178200 | 8 | 27868323:27871541 | 560 | 1683 | 6.45 | 61206.56 | 59.8 | 60.84 |
| VabZIP48 | KOM51505 | Vigan09g016400 | 9 | 2293402:2297134 | 414 | 1245 | 9.76 | 44836.57 | 51.3 | 63.33 |
| VabZIP49 | KOM51608 | Vigan09g026700 | 9 | 3077698:3078135 | 145 | 438 | 8.39 | 16818.88 | 66.44 | 69.24 |
| VabZIP50 | KOM51681 | Vigan09g034000 | 9 | 3752763:3754756 | 173 | 522 | 8.96 | 19114.95 | 81.03 | 60.35 |
| VabZIP51 | KOM52069 | Vigan09g072800 | 9 | 9572258:9574461 | 401 | 1206 | 9.53 | 44184.35 | 49.5 | 66.66 |
| VabZIP52 | KOM52141 | Vigan09g080000 | 9 | 10781336:10784902 | 317 | 954 | 5.28 | 34486.59 | 51.7 | 53 |
| VabZIP53 | KOM52631 | Vigan09g129000 | 9 | 17690389:17691494 | 192 | 579 | 7.95 | 21957.66 | 65.1 | 70.68 |
| VabZIP54 | KOM52710 | Vigan09g136900 | 9 | 18563364:18566199 | 383 | 1152 | 5.86 | 41300.38 | 53.85 | 58.72 |
| VabZIP55 | KOM54030 | Vigan09g268900 | 9 | 31150096:31151700 | 160 | 483 | 9.45 | 18058.57 | 69.09 | 84.06 |
| VabZIP56 | KOM54352 | Vigan10g024400 | 10 | 2200602:2203545 | 773 | 2322 | 5.47 | 84105.11 | 49.86 | 66.66 |
| VabZIP57 | KOM54790 | Vigan10g068200 | 10 | 7479252:7480553 | 274 | 825 | 8.96 | 30187.76 | 47.09 | 77.26 |
| VabZIP58 | KOM55086 | Vigan10g097800 | 10 | 11943045:11944253 | 226 | 681 | 7.85 | 24910.04 | 64.85 | 69.87 |
| VabZIP59 | KOM55415 | Vigan10g130700 | 10 | 17670459:17670974 | 171 | 516 | 10.01 | 19365.09 | 58.74 | 80.41 |
| VabZIP60 | KOM55637 | Vigan10g152900 | 10 | 21362677:21364733 | 366 | 1101 | 8.87 | 40250.02 | 63.93 | 67.4 |
| VabZIP61 | KOM55719 | Vigan10g161100 | 10 | 22156563:22160320 | 425 | 1278 | 6.54 | 45837.57 | 57.69 | 58.59 |
| VabZIP62 | KOM56298 | Vigan10g219000 | 10 | 31906566:31907424 | 250 | 753 | 7.76 | 27885.32 | 40.53 | 76 |
| VabZIP63 | KOM56416 | Vigan10g230800 | 10 | 33431731:33432333 | 200 | 603 | 6.35 | 23381.72 | 77.42 | 60.45 |
| VabZIP64 | KOM57378 | Vigan11g041100 | 11 | 5107747:5108058 | 103 | 312 | 10.36 | 12782.64 | 59.7 | 65.34 |
| VabZIP65 | KOM58023 | Vigan11g105600 | 11 | 16199010:16201601 | 164 | 495 | 7.77 | 18419.76 | 33.37 | 72.07 |
| VabZIP66 | KOM27806 | Vigan464s000300 | Un | 35436:36353 | 212 | 639 | 5.9 | 22932.73 | 53.69 | 82.78 |
| VabZIP67 | KOM26299 | Vigan252s001100 | Un | 103219:103617 | 132 | 399 | 8.77 | 14997.97 | 66.99 | 87.05 |
| VabZIP68 | KOM29371 | Vigan661s000800 | Un | 125891:128008 | 119 | 360 | 10.92 | 13509.2 | 53.02 | 59.83 |
| VabZIP69 | KOM25975 | Vigan211s001000 | Un | 132366:132776 | 136 | 411 | 7.85 | 15403.4 | 68.76 | 83.09 |
| VabZIP70 | KOM29488 | Vigan707s002000 | Un | 318623:321499 | 400 | 1203 | 6.04 | 42880.65 | 50.76 | 62.25 |
| VabZIP71 | KOM28102 | Vigan499s004600 | un | 506479:507261 | 201 | 606 | 8.74 | 22585.54 | 56.47 | 68.01 |
| VabZIP72 | KOM28686 | Vigan561s007600 | Un | 865793:870130 | 293 | 882 | 5.78 | 32179.67 | 42.86 | 65.56 |
