# Supplementary material for: The bZIP Transcription Factor Family in Adzuki Bean (Vigna Angularis): Genome-Wide Identification, Evolution, and Expression Under Abiotic Stress During the Bud Stage
Source: Front Genet. 2022 Apr 25;13:847612. doi: 10.3389/fgene.2022.847612 (PMC9081612; doi:10.3389/fgene.2022.847612)
Supplement: Supplementary file 7 [file Table4.DOCX]

Table S4: Predicted functions of elements.

| *Cis*-elements | The predicted function of elements |
| --- | --- |
| ABRE | *Cis-*acting element involved in the abscisic acid responsiveness. |
| ARE | *Cis-*acting regulatory element essential for the anaerobic induction. |
| TATC-box | *Cis-*acting element involved in gibberellin-responsiveness. |
| LTR | *Cis-*acting element involved in low-temperature responsiveness. |
| TCA-element | *Cis-*acting element involved in salicylic acid responsiveness. |
| GC-motif | Enhancer-like element involved in anoxic specific inducibility. |
| MBS | MYB binding site involved in drought-inducibility. |
| GARE-motif | Gibberellin-responsive element. |
| P-box | Gibberellin-responsive element. |
| RY-element | *Cis*-acting regulatory element involved in seed-specific regulation. |
| MBSI | MYB binding site involved in flavonoid biosynthetic genes regulation. |
| NON-box | *Cis*-acting regulatory element related to meristem specific activation. |
